# Mapping artificial intelligence adoption in hepatology practice and research: challenges and opportunities in MENA region

**DOI:** 10.3389/fmed.2025.1630831

**Published:** 2025-09-12

**Authors:** Mohamed El-Kassas, Rofida Khalifa, Mohammed A. Medhat, Yusuf Yilmaz, Ali Tumi, Asma Labidi, Maen Almattooq, Faisal M. Sanai, Mohamed Elbadry, Mohammed Omer Mohammed, Mohamed Abdelhakim Mahdy, Nermeen Abdeen, Hend Radwan, Walaa Abdelhamed, Majed Almaghrabi, Mai Fared, Abdel-Naser Elzouki, Khalid A. Alswat, Khalid M. AlNaamani, the Steatotic Liver Disease Study Foundation in Middle East and North Africa (SLMENA) Collaborators, Heba Shafeak Abd El Khalik

**Affiliations:** ^1^Department of Endemic Medicine, Faculty of Medicine, Helwan University, Cairo, Egypt; ^2^Applied Science Research Center, Applied Science Private University, Amman, Jordan; ^3^Steatotic Liver Diseases Study Foundation in Middle East and North Africa (SLMENA), Cairo, Egypt; ^4^Department of Tropical Medicine, Faculty of Medicine, Minia University, Minya, Egypt; ^5^Department of Tropical Medicine and Gastroenterology, Assiut University, Assiut, Egypt; ^6^Department of Gastroenterology, School of Medicine, Recep Tayyip Erdoğan University, Rize, Türkiye; ^7^Department of Medical, Central Hospital, Tripoli, Libya; ^8^Department of Gastroenterology “A”, Rabta Hospital, Tunis, Tunisia; ^9^Department of Gastroenterology and Hepatology, Jaber AlAhmad Hospital, Kuwait City, Kuwait; ^10^Gastroenterology Section, Department of Medicine, King Abdulaziz Medical City, King Abdullah International Medical Research Center, Ministry of National Guard – Health Affairs, Jeddah, Saudi Arabia; ^11^Department of Clinical Science, University of Sulaimani, Sulaimani, Iraq; ^12^Gastroenterology and Hepatology Unit, Assiut University, Assiut, Egypt; ^13^Department of Tropical Medicine, Faculty of Medicine, Alexandria University, Alexandria, Egypt; ^14^Department of Internal Medicine, Medicine and Clinical Studies Research Institute, National Research Center, Cairo, Egypt; ^15^Department of Tropical Medicine and Gastroenterology, Faculty of Medicine, Sohag University, Sohag, Egypt; ^16^Gastroenterology Section, Department of Medicine, King, Abdulaziz Medical City, Jeddah, Saudi Arabia; ^17^King Abdullah International Medical Research Center and College of Medicine, King Saud bin Abdulaziz University for Health Sciences, Jeddah, Saudi Arabia; ^18^Research Support Center, Helwan University, Cairo, Egypt; ^19^Department of Medicine, Hamad Medical Corporation and College of Medicine, Qatar University, Doha, Qatar; ^20^Liver Disease Research Center, Department of Medicine, College of Medicine, King Saud University, Riyadh, Saudi Arabia; ^21^Division of Gastroenterology and Hepatology, Department of Medicine, The Medical City for Military and Security Services, Muscat, Oman

**Keywords:** artificial intelligence (AI), hepatology, MENA region, medical education, ethical considerations, digital health

## Abstract

**Background:**

Artificial intelligence (AI) is increasingly relevant to hepatology, yet real-world adoption in the Middle East and North Africa (MENA) is uncertain. We assessed awareness, use, perceived value, barriers, and policy priorities among hepatology clinicians in the region.

**Methods:**

A cross-sectional online survey targeted hepatologists and gastroenterologists across 17 MENA countries. The survey assessed clinical and research applications of AI, perceived benefits, clinical and research use, barriers, ethical considerations, and institutional readiness. Descriptive statistics and thematic analysis were performed.

**Results:**

Of 285 invited professionals, 236 completed the survey (response rate: 82.8%). While 73.2% recognized the transformative potential of AI, only 14.4% used AI tools daily, primarily for imaging analysis and disease prediction. AI tools were used in research by 39.8% of respondents, mainly for data analysis, manuscript writing assistance, and predictive modeling. Major barriers included inadequate training (60.6%), limited AI tool access (53%), and insufficient infrastructure (53%). Ethical concerns focused on data privacy, diagnostic accuracy, and over-reliance on automation. Despite these challenges, 70.3% expressed strong interest in AI training., and 43.6% anticipating routine clinical integration within 1–3 years.

**Conclusion:**

MENA hepatologists are optimistic about AI but report limited routine use and substantial readiness gaps. Priorities include scalable training, interoperable infrastructure and standards, clear governance with human-in-the-loop safeguards, and region-specific validation to enable safe, equitable implementation.

## 1 Introduction

Over the past two decades, hepatology practice has witnessed significant advancements in diagnostic, prognostic, and therapeutic fields. The integration of artificial intelligence (AI) into hepatology holds the potential to further transform the evaluation of complex clinical data, with AI applications demonstrating capabilities that, in specific tasks, surpass those of physicians (1, 2). In hepatology, AI has been utilized in areas such as liver imaging, histopathology interpretation, non-invasive testing, and evidence-based decision-making (1, 3, 4). It also enhances the analysis of large datasets, facilitating the advancement of precision medicine (5), and improving the efficiency and cost-effectiveness of telemedicine while supporting personalized, evidence-based care (6).

Specifically, AI applications have shown promise in identifying liver fibrosis, distinguishing different types of liver lesions, forecasting outcomes of chronic liver conditions, and suggesting management of metabolic- dysfunction steatotic liver disease (MASLD) (7). AI technologies have also been investigated for their potential to improve the diagnosis, prognosis, and treatment of hepatocellular carcinoma (HCC), a major challenge in hepatology (8).

Despite these advancements, the implementation of AI in hepatology faces several challenges, including data heterogeneity, ethical considerations, system interoperability, and the necessity for inclusive datasets that represent diverse populations (4). Issues related to data collection, standardization, and interpretation further hinder the widespread adoption of AI tools. Algorithms that exhibit deficiencies due to concerns related to data privacy and quality may pose potential risks when applied in clinical settings (3).

In the Middle East and North Africa (MENA) region, the adoption of AI in hepatology and gastroenterology has been relatively slow (9). This delay may be attributed to a lack of awareness, technical capacity, regulatory guidelines, and concerns regarding data privacy and clinical reliability. Understanding the perspectives of hepatologists on AI is crucial for addressing these challenges and effectively integrating AI-generated tools into clinical practice. Accordingly, we assessed hepatologists’ familiarity with AI, usage patterns, and concerns about its integration into practice to inform strategies for adoption across the MENA region. This work addresses a specific gap not covered by prior surveys in other regions and specialties (9–15): it is the first multinational assessment focused specifically on hepatologists from 17 MENA countries; it evaluates clinical and research use of AI alongside institutional readiness; and it integrates quantitative findings with a thematic synthesis of policy-level recommendations.

## 2 Materials and methods

### 2.1 Study design and participants

This study utilized a cross-sectional survey design to evaluate hepatologists’ perceptions, awareness, and practical applications of AI across the MENA region. The target population included physicians specializing in hepatology, gastroenterology, and internal medicine who are involved in liver disease management within the MENA region. Data collection took place between April and May 2025.

### 2.2 Survey instrument and pilot testing

The survey was collaboratively developed by a multidisciplinary team of hepatologists and researchers with expertise in medical education, digital health, and AI applications in clinical practice. Items were developed from a targeted review of surveys on clinician AI adoption and digital health readiness (10–15) and aligned with widely cited principles for safe clinical AI deployment (4). The final instrument comprised 37 items across six domains—demographics/professional profile (11), clinical exposure and utilization of AI (3), perceptions/attitudes/trust (10), institutional readiness/barriers/ethics (5), research use and educational needs (6), and two open-ended questions capturing policy-oriented recommendations and additional comments. Response formats included single- or multiple-choice items, 5-point frequency scales (never to daily), and 0–10 rating scales (e.g., perceived potential, institutional willingness). The survey instrument was hosted on the secure SurveyMonkey platform. Prior to deployment, the survey underwent pilot testing with 10 participants to assess clarity, content relevance, and technical functionality. This sample size is commonly used for cognitive debriefing/pretesting in survey design, enabling item-level refinement without imposing substantive respondent burden. The pilot group intentionally spanned career stages (consultants/specialists/fellows) and practice settings (academic and public), and included participants from 5 countries across North Africa and the Gulf (Egypt, Türkiye, Oman, Saudi Arabia, Tunisia). Content validity was assessed qualitatively via expert review by hepatologists involved in medical education and digital health, followed by pilot debriefing to confirm item relevance and coverage. Feedback was collected using a structured comment form embedded at the end of the pilot survey and brief follow-up calls for clarification. Minor revisions included wording simplification (e.g., defining MASLD/MASH on first mention), response-option reordering (ascending frequency), addition of a “not applicable/prefer not to answer” option where relevant, and layout adjustments to improve item flow.

### 2.3 Survey distribution and sampling strategy

The finalized survey was distributed via multiple channels, including direct email invitations and dedicated WhatsApp groups targeting medical professionals. The extensive network of the Steatotic Liver Diseases Study Foundation in the Middle East and North Africa (SLMENA) was leveraged to enhance regional outreach. A snowball sampling technique was employed, encouraging initial respondents to share the survey with colleagues to broaden the sample. Initial seed contacts were identified purposively through the SLMENA network to include clinicians from multiple MENA subregions (North Africa, Levant, Gulf) and practice sectors (academic/public), after which snowball sharing expanded reach. To mitigate over-representation, we monitored country and sector distribution weekly and issued targeted reminders to under-represented groups via SLMENA channels.

### 2.4 Survey content and data management

The survey included both closed-ended questions (using Likert-scale responses) and open-ended items, covering domains such as clinical and research applications of AI, perceived benefits and barriers, ethical considerations, and implementation challenges. Participants were given the option to provide their names for acknowledgment purposes in the resulting publication. All data were anonymized, stored securely, accessed only by the research team, and used solely for research purposes. The final instrument comprised 37 items organized into the following domains: (1) demographics and professional profile (11 items); (2) clinical exposure and utilization of AI (3 items); (3) perceptions, attitudes, and trust (10 items); (4) institutional readiness, barriers, and ethics (5 items); (5) research use of AI and educational needs (6 items); and (6) two open-ended questions capturing policy-oriented recommendations and additional comments.

The full questionnaire is provided in Supplementary File S1, and the qualitative codebook with exemplar quotations is provided in Supplementary File S2.

### 2.5 Ethical considerations

The study was approved by the Research Ethics Committee, Faculty of Medicine, Helwan University, Egypt (Serial: 58-2025) and conducted in accordance with the Declaration of Helsinki. Participation was voluntary; electronic informed consent was obtained before any survey items were displayed. The consent page described the study aims, procedures, minimal risks, data use, confidentiality safeguards, and the right to decline or withdraw at any time without consequences. Respondents could optionally provide their names solely for acknowledgment purposes; analytical datasets were otherwise de-identified. Data were stored on password-protected, access-restricted servers and are reported only in aggregate to preserve confidentiality.

### 2.6 Statistical data analysis

Survey responses were exported from Microsoft Excel and analyzed using IBM SPSS Statistics version 26.0 (SPSS Inc., Chicago, IL, United States). Descriptive statistics (frequencies and percentages) summarized categorical variables. Given the non-probability (convenience/snowball) sampling and small cell sizes for several strata, we prespecified a descriptive analytic strategy and did not conduct formal hypothesis testing or model-based inference, to avoid overstating generalizability. Open-ended responses were analyzed using an inductive, semantic thematic analysis (six-step approach: familiarization; generating initial codes; searching, reviewing, defining/naming themes; reporting). Two researchers (RK and MAM) independently coded the dataset, reconciled discrepancies by consensus (analyst triangulation), and refined a shared codebook managed in Microsoft Excel. Themes are summarized in Supplementary Table S1; the final codebook with anonymized exemplar quotations is provided in Supplementary File S2.

## 3 Results

We received 236 completed responses that met inclusion criteria. Based on tracked seed and first-wave invitations (*n* = 285) disseminated via SLMENA channels, the estimated participation rate was 82.8%; because downstream snowball sharing was not fully traceable, a conventional response rate cannot be calculated. Participants were predominantly male (65.3%). The age distribution was balanced, with the largest groups being those aged 36–45 years (31.4%), followed by ages 25–35 (28.0%) and 46–55 years (23.7%).

The most common specialties reported were hepatology and gastroenterology, each accounting for 33.9% of participants. The respondents represented 17 MENA countries, primarily Turkiye (27.1%), Egypt (24.6%), and Oman (15.3%). Participants largely worked in their countries of origin, with minimal exceptions. Most respondents worked at academic or teaching hospitals (66.1%), followed by public hospitals (26.7%), with a significant majority serving as clinicians (91.9%).

**Figure 1:**
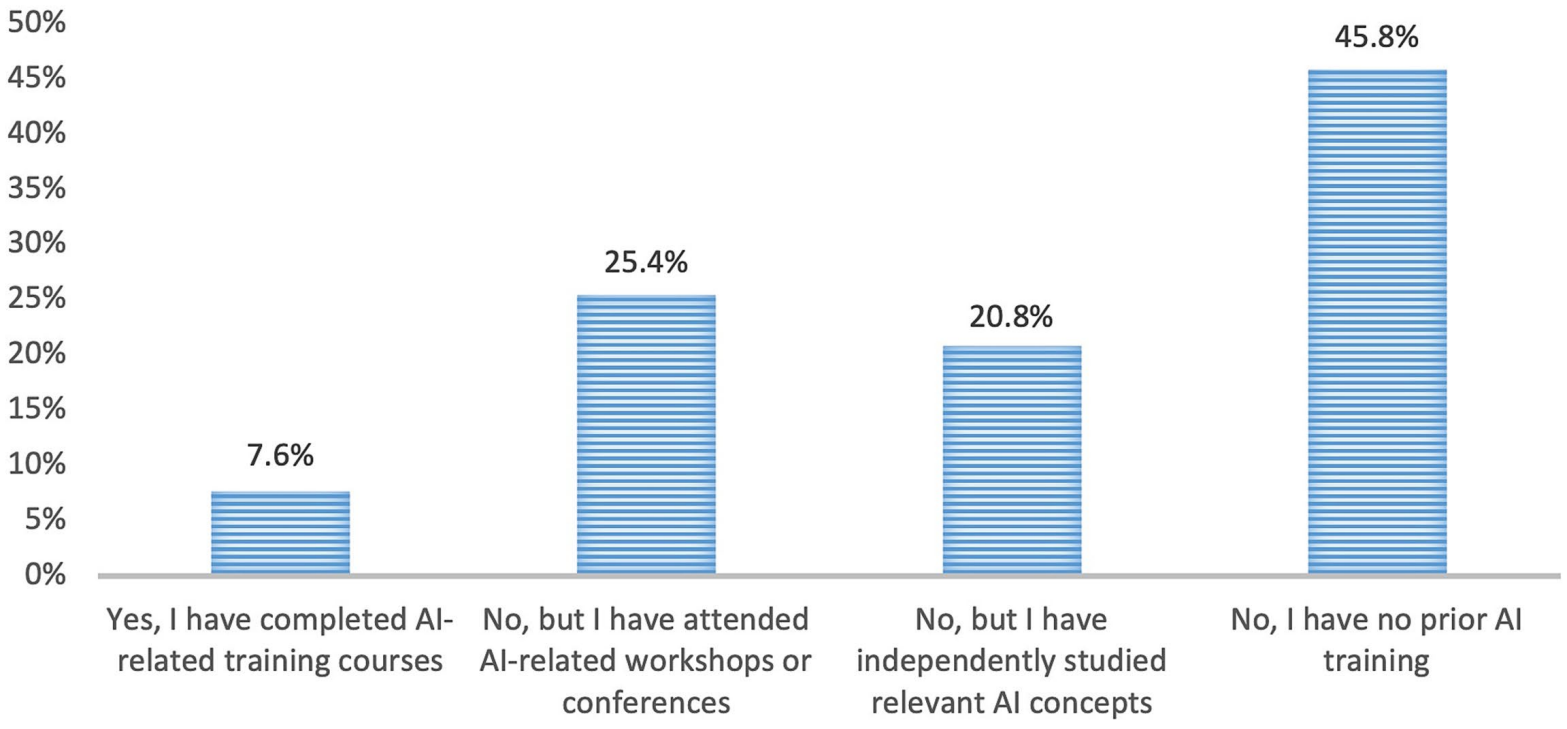
Distribution of previous formal training in AI applications in medicine.

Research activity among participants was moderate, with over 60% reporting between 1 and 5 peer-reviewed hepatology publications, and 20.3% having 6–25 publications. Professional experience was diverse, with 26.3% reporting over 20 years of practice and 28.0% having less than 5 years. Despite growing interest in AI, formal education in AI was notably limited; only 7.6% had completed formal AI training, and 45.8% had no prior AI education or exposure (Table 1; Figure 1).

**Table 1:** Demographic characteristics, professional background, and AI training exposure of hepatologists in the MENA region.

Variables	*n* = 236	%
Gender		
Female	82	34.7
Male	154	65.3
Age (years)		
25–35	66	28.0
36–45	74	31.4
46–55	56	23.7
56–65	32	13.6
> 65	8	3.4
Primary specialty		
Hepatology	80	33.9
Gastroenterology	80	33.9
Internal Medicine	41	17.4
Transplant Hepatology	15	6.4
Other^a^	20	8.5
Country (Nationality)		
Turkey	64	27.1
Egypt	58	24.6
Oman	36	15.3
Saudi Arabia	22	9.3
Libya	14	5.9
Iraq	12	5.1
Qatar	6	2.5
Kuwait	4	1.7
Tunisia	4	1.7
Algeria	3	1.3
Other^b^	13	5.5
Country of work		
Turkey	65	27.5
Egypt	57	24.2
Oman	37	15.7
Saudi Arabia	23	9.7
Libya	15	6.4
Iraq	11	4.7
Qatar	6	2.5
Kuwait	4	1.7
Tunisia	4	1.7
Algeria	3	1.3
Other^c^	11	4.7
Primary sector of work		
Academia (University/Teaching Hospital)	156	66.1
Public Hospital	63	26.7
Private Practice	11	4.7
Research Institution	3	1.3
Other^d^	3	1.3
Primary field or area of work		
Clinician/Medical Doctor	217	91.9
Clinical Research	9	3.8
Healthcare Administration	5	2.1
Education/Pedagogy	2	0.8
Other^e^	3	1.3
Number of peer-reviewed publications in hepatology		
1–5	145	61.4
6–25	48	20.3
26–50	24	10.2
51–100	11	4.7
>100	8	3.4
Years of experience managing patients with liver diseases		
Less than 5 years	66	28.0
5–10 years	54	22.9
11–20 years	54	22.9
More than 20 years	62	26.3

a Other: Dietitian/Nutritionist (*n* = 7), Family medicine (*n* = 4), Cardiology (*n* = 2), Tropical medicine (*n* = 2), Diving and hyperbaric medicine (*n* = 2), General surgery, Critical care medicine, Endocrinology (*n* = one each).

b Other: Bahrain, Jordan, Morocco, Yemen (*n* = 2 each) and Palestine, United Arab Emirates, Lebanon, USA, UK (*n* = one each).

c Other: Bahrain, Jordan, Morocco, Yemen (*n* = 2 each), and Palestine, United Arab Emirates, Lebanon (*n* = one each).

d Other: Primary health care, Non-profit organization, Tertiary center (*n* = one each).

e Other: Clinical dietitian, Nutritionist, Non-clinical research (*n* = one each).

Experience categories reflect pre-specified career-stage bands collected in the survey (<5, 6–10, 11–20, >20 years). To preserve the integrity of reported frequencies, categories were not re-binned post hoc.

### 3.1 AI utilization in clinical hepatology practice

Among participants, 30.5% reported never using AI-based tools in clinical practice, and 26.3% used them only rarely. Conversely, daily AI use was reported by 14.4%, with weekly and monthly use at 18.6 and 10.2%, respectively.

**Figure 2:**
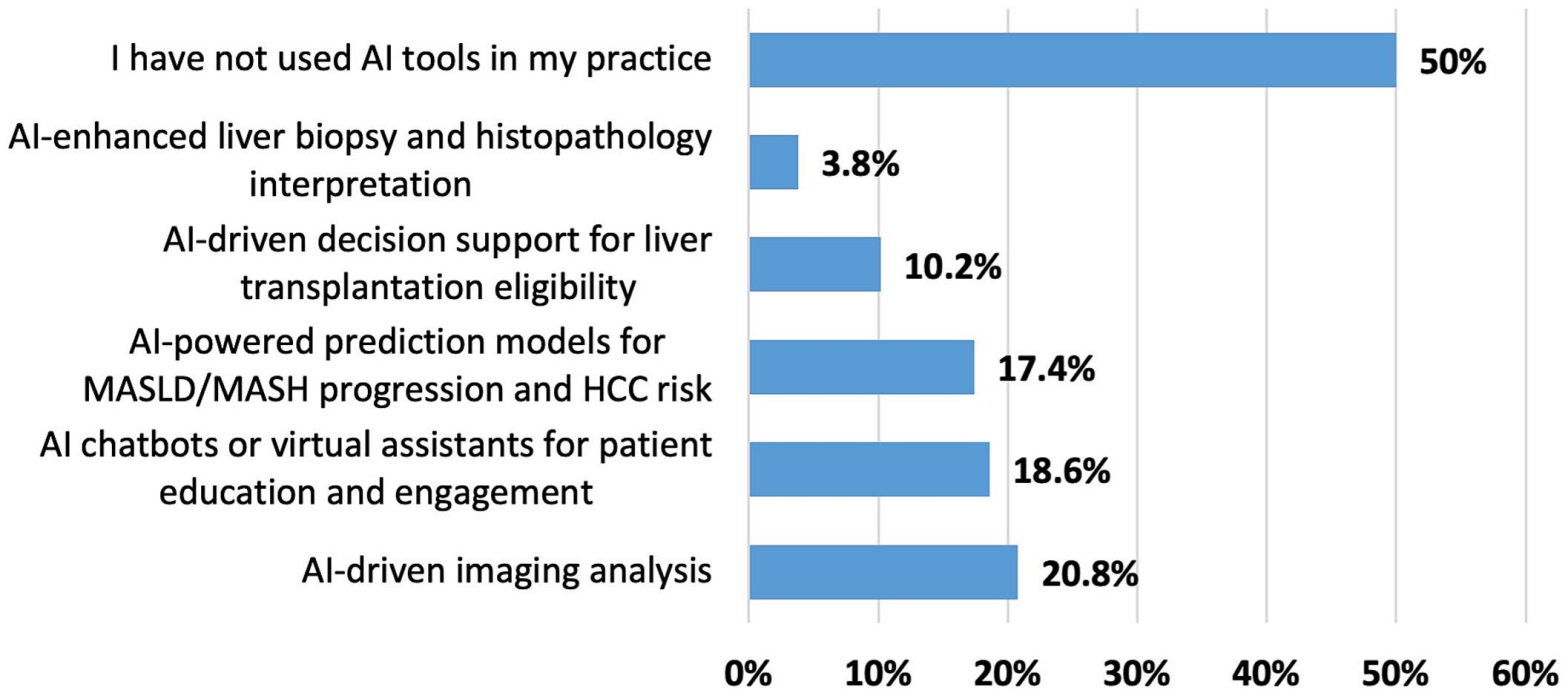
Types of AI tool utilization in clinical hepatology practice in the MENA region.

The most frequently utilized AI applications included AI-driven imaging analysis (20.8%), AI-powered chatbots or virtual assistants for patient education (18.6%), and predictive models for MASLD progression or HCC risk (17.4%). Less frequently used were AI-supported decision-making tools for assessing liver transplantation eligibility (10.2%) and AI-enhanced histopathological interpretation of liver biopsies (3.8%). Notably, half of the respondents indicated no use of any AI tools (Figure 2).

### 3.2 Perceptions and attitudes towards AI in hepatology clinical practice

Most respondents expressed optimism about AI’s potential in hepatology. On a 0–10 Likert scale, 73.2% rated its transformative potential of AI between 7 and 10, with scores of 8 (25.8%), 7 (20.3%), and 10 (16.1%) being the most common. Only 6.3% rated AI below 5. The leading perceived benefit was the role of AI in early diagnosis and disease detection (46.2%), followed by risk stratification and prognosis prediction (30.5%). Fewer respondents prioritized administrative automation (12.7%), treatment planning (5.5%), or patient education (5.1%).

Regarding patient outcomes, 29.7% of respondents anticipated a highly positive impact, while 41.5% expected a somewhat positive effect. Only 8.9% were pessimistic, mainly due to concerns about reliability and diagnostic accuracy.

When asked about improving access to care, 36.0% believed AI could significantly reduce disparities, while 50.8% felt it could offer modest improvements. However, 9.3% perceived no benefit, and 3.8% cautioned that AI could worsen access inequalities due to uneven technology distribution.

Views on cost-effectiveness were mixed: 47.9% of respondents believed AI could reduce healthcare costs by improving efficiency, while 36% were uncertain and 16.1% expected increased costs.

Trust in AI remained modest. Most respondents (57.6%) supported AI use only with human validation, and just 6.8% expressed high trust. Consistently, 75% were comfortable relying on AI if expert oversight was ensured, while only 8.1% endorsed fully autonomous decision-making.

As for patient education, 52.1% of respondents preferred AI to support, rather than to replace, human interaction. Only 26.3% favored AI as the primary tool for delivering personalized education (Table 2).

**Table 2:** Perceptions of AI’s potential, impact, and trust among hepatologists in the MENA region.

Variables	*n* = 236	%
To what extent do you believe AI can enhance hepatology practice? [Likert Scale] 0 (No enhancement) to 10 (Highly transformative)		
0	1	0.4
2	2	0.8
3	9	3.8
4	3	1.3
5	25	10.6
6	23	9.7
7	48	20.3
8	61	25.8
9	26	11.0
10	38	16.1
Rank areas of hepatology you think AI can contribute to most *		
Diagnosis and early detection of liver diseases	109	46.2
Risk stratification and prognosis prediction	72	30.5
Automating administrative tasks (e.g., patient scheduling, documentation)	30	12.7
Treatment planning and decision support	13	5.5
Enhancing patient education and adherence	12	5.1
How do you perceive the impact of AI on patient outcomes in hepatology?		
Negative impact – AI may increase errors or misdiagnosis	5	2.1
Somewhat negative impact – AI may not be reliable in clinical settings	16	6.8
Neutral – AI impact is uncertain	47	19.9
Somewhat positive impact – AI may assist in some areas but is not essential	98	41.5
Highly positive impact – AI will significantly improve hepatology practice	70	29.7
In your opinion, will AI improve access to hepatology care in underserved areas?		
Yes, significantly – AI can bridge the gap in resource-limited settings	85	36.0
Somewhat – AI can assist but may not fully replace hepatologists	120	50.8
No impact – AI will not change accessibility issues	22	9.3
May worsen disparities – AI access may be limited to wealthier regions	9	3.8
Do you think AI will contribute to reducing healthcare costs in hepatology?		
Yes, AI can streamline workflows and reduce unnecessary procedures	113	47.9
No, AI implementation may introduce additional costs	38	16.1
Uncertain, AI’s cost-effectiveness depends on the healthcare system	85	36.0
How much do you trust AI-based decisions in hepatology compared to human expertise?		
No trust at all – AI is unreliable in hepatology	7	3.0
Limited trust – AI should always be secondary to human decisions	77	32.6
Moderate trust – AI is useful but needs human validation	136	57.6
High trust – AI can make accurate recommendations comparable to human experts	16	6.8
Would you feel comfortable relying on AI-driven recommendations in critical hepatology cases?		
Yes, AI should be used as an independent decision-making tool	19	8.1
Yes, but only when combined with human validation	177	75.0
No, AI should not be used in critical decision-making	40	16.9
Do you believe AI should have a role in patient counseling and education in hepatology?		
Yes, AI can provide personalized education and support for patients	62	26.3
No, human interaction is essential for effective patient counseling	38	16.1
AI can assist but should not replace human involvement	123	52.1
Uncertain, AI’s role in counseling needs further research	13	5.5

* Questions with multiple responses.

### 3.3 Perceived readiness, institutional adoption, and priority areas for AI implementation

Although only 6.8% of respondents reported that AI is currently well-integrated in their institutions, a much larger group (43.6%) anticipated routine use of AI in hepatology practice within 1–3 years, and 27.1% expected adoption within 4–7 years.

Institutional willingness to adopt AI, assessed via a 0–10 Likert scale, showed a broad range of responses, with the most common ratings being 5 (20.3%), 6 (13.6%), and 7 (11.4%), reflecting moderate but cautious readiness levels.

**Figure 3:**
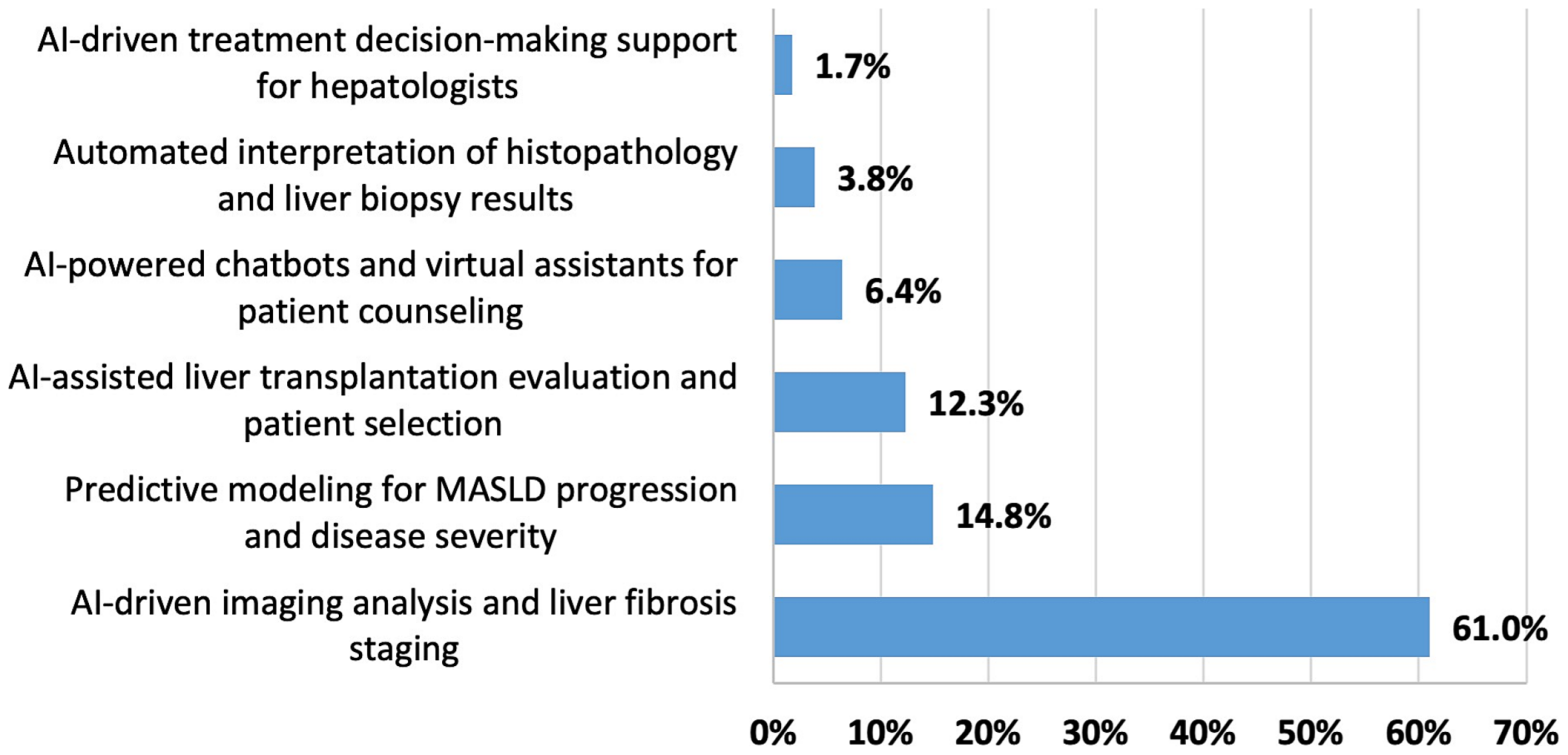
Expert-driven prioritization of AI applications in hepatology clinical practice.

When asked to prioritize areas for AI integration, most respondents (61.0%) favored AI-driven imaging analysis and clinically staging of liver fibrosis. This was followed by predictive modeling for MASLD progression (14.8%) and AI-assisted liver transplant evaluations (12.3%). Other applications such as patient counseling chatbots (6.4%), automated histopathology interpretation (3.8%), and AI-guided treatment decisions (1.7%) were less commonly prioritized (Table 3; Figure 3).

**Table 3:** Institutional readiness and preferred areas for AI implementation in hepatology practice among hepatologists in the MENA region.

Variables	*n* = 236	%
How soon do you think AI will become a routine part of hepatology practice in your institute?		
Already widely used	16	6.8
Within 1–3 years	103	43.6
Within 4–7 years	64	27.1
More than 7 years	18	7.6
Uncertain	35	14.8
How would you rate the willingness of your institution to implement AI in hepatology? [Likert Scale] 0 (Not willing at all) to 10 (Very willing)		
0	7	3.0
1	13	5.5
2	24	10.2
3	23	9.7
4	9	3.8
5	48	20.3
6	32	13.6
7	27	11.4
8	19	8.1
9	11	4.7
10	23	9.7

### 3.4 Barriers, ethical concerns, and proposed solutions for AI adoption in hepatology practice

As summarized in Table 4, the most commonly reported barrier to AI adoption was the lack of technical expertise and specialized training among hepatologists cited by (60.6%) of respondents. This was followed by limited access to AI tools and inadequate infrastructure (each cited by 53% of respondents), along with financial constraints related to high technology costs (46.6%). Regulatory and integration challenges were also frequent, including the absence of standardized frameworks reported by (44.1%) and lack of integration with electronic medical records reported by (41.9%) of the respondents. Additional concerns included ethical issues, limited collaboration between stakeholders, and resistance to change within the profession.

**Table 4:** Barriers, concerns, and ethical issues for AI adoption in hepatology: perspectives of hepatologists in the MENA region.

Variables	*n* = 236	%
What are the biggest barriers to AI adoption in hepatology practice in your institute? *		
Lack of technical expertise and specialized training for hepatologists	143	60.6
Limited access to AI-driven diagnostic tools and software	125	53.0
Insufficient AI infrastructure in hospitals and research institutions	125	53.0
High cost of AI technology and infrastructure	110	46.6
Lack of standardized regulatory frameworks for AI implementation in healthcare	104	44.1
Lack of integration with electronic medical records (EMRs) and hospital systems	99	41.9
Ethical concerns regarding AI use in medical decision-making	86	36.4
Limited collaboration between AI developers and hepatologists	82	34.7
Resistance to change among healthcare professionals	76	32.2
Poor responsiveness of AI models to new and emerging data and paradigms	24	10.2
Lack of adaptability to tailor to patient needs	23	9.7
Other^a^	2	0.8
What ethical concerns do you have about AI implementation in hepatology? *		
Data security and patient confidentiality risks	134	56.8
AI bias and errors leading to misdiagnosis	131	55.5
Over-reliance on AI and reduced clinical judgment by physicians	118	50.0
Ethical concerns about AI replacing human expertise in hepatology	101	42.8
Liability issues in AI-assisted medical decision-making	86	36.4
Lack of evidence of the benefits of Al-generated information	85	36.0
Lack of transparency in AI decision-making models	72	30.5
Other^b^	1	0.4
Rank your biggest concern regarding AI integration in hepatology *		
Accuracy and reliability of AI-driven diagnostics	118	50.0
Data privacy and ethical concerns	55	23.3
Dependence on AI leading to reduced clinical skills	43	18.2
Cost and financial barriers	11	4.7
Regulatory and legal issues	9	3.8
What steps should be taken to enhance trust in AI-based hepatology solutions? *		
Developing clear regulatory guidelines for AI in healthcare	178	75.4
Increasing hepatologists’ training on AI applications	167	70.8
Conducting clinical trials to validate AI effectiveness in hepatology	140	59.3
Encouraging collaborations between AI developers and medical professionals	134	56.8
Ensuring AI models are transparent and interpretable	117	49.6
Implementing AI-based decision support systems with real-time feedback	104	44.1
Other^c^	3	1.3

* Questions with multiple responses.

a Other: Lack of awareness for what AI means in hepatology and how it can help, Niche developments and applications, the logical process of neural networks in AI.

b Other: AI mechanisms always try to make judgments based on objective algorithms when considering diagnosis and treatment options, but in real life there are always patients who remain in the “gray zone”.

c Other: Create AI monitoring unit, technical support for integration, More training courses.

Ethical concerns were particularly pronounced. Respondents highlighted risks related to data security and confidentiality (56.8%), algorithmic bias and potential misdiagnosis (55.5%), and over-reliance on AI undermining clinical judgment (50%). Others expressed fears of AI replacing human expertise (42.8%) and uncertainty around medico-legal liability in AI-supported decisions (36.4%).

When asked about their main concern, half of the participants cited the accuracy and reliability of AI diagnostics. This was followed by concerns related to privacy and broader ethical implications reported by 23.3%, and the impact of AI on clinical skill development reported by 18.2% of respondents.

To address these issues and foster trust, participants highlighted several key strategies: establishing clear regulatory guidelines (75.4%), providing targeted education on AI (70.8%), and clinically validating AI tools (59.3%). Additional priorities included promoting collaboration between developers and clinicians (56.8%) and enhancing transparency and interpretability of AI models (49.6%).

### 3.5 AI in research and training

Around 40% of respondents reported using AI-powered tools in research, with 13.6% using them frequently and 28.4% occasionally. Meanwhile, 51.7% expressed interest in exploring AI for research purposes, despite no prior use. Only 6.4% believed AI was unnecessary in research.

Among users, the most common applications of AI were literature review and summarization (42%), AI-driven statistical analysis (27.1%), and manuscript writing assistance (26.7%). Less frequent reported uses included predictive modeling and AI-based image processing. Notably, 58.1% indicated they had not used any AI tools for research.

Concerns were prevalent: 66.1% cited issues related to accuracy and reliability, 55.1% highlighted the use of unvalidated methods, and 52.1% raised ethical concerns regarding AI use in manuscript drafting. Nearly half (46.2%) were also concerned about AI bias potentially distorting research conclusions.

Despite these concerns, most acknowledged AI’s value: 37.3% believed it could significantly enhance research efficiency and quality, while 55.9% supported its use with human oversight. Only 2.1% were opposed and 4.7% were uncertain.

Interest in AI training was high, 70.3% were eager to attend workshops, and 28.8% were open to training if relevant to their work. The preferred educational formats included hands-on workshops and simulations (73.3%), formal courses and certifications (65.7%), and online platforms (61.4%). More than half of the participants supported integrating AI into medical curricula and collaborating with AI experts (Table 5).

**Table 5:** Utilization of AI in hepatology research and training: experiences, perceptions, and educational needs among hepatologists in the MENA region.

Variables	*n* = 236	%
Have you used AI-powered tools in your research work?		
Yes, frequently	32	13.6
Yes, occasionally	67	28.4
No, but I am interested in using them	122	51.7
No, I do not see a need for AI in research	15	6.4
Which AI-based applications have you used in your hepatology research? *		
AI-assisted literature review and summarization	99	42.0
AI-powered statistical analysis and data interpretation	64	27.1
AI-based manuscript writing assistance	63	26.7
AI-driven predictive modeling for hepatology studies	41	17.4
AI-driven image processing for histopathology and imaging studies	32	13.6
Not applicable (Not used yet)	137	58.1
Other^a^	1	0.4
What is your primary concern regarding AI use in research and publication? *		
Accuracy and reliability of AI-generated results	156	66.1
Lack of proper validation for AI-driven research methodologies	130	55.1
Ethical concerns regarding AI-assisted manuscript writing	123	52.1
AI bias leading to incorrect conclusions	109	46.2
Do you think AI can improve the efficiency and quality of research in hepatology?		
Yes, significantly	88	37.3
Somewhat, but with human oversight	132	55.9
No, traditional methods are more reliable	5	2.1
Uncertain	11	4.7
Would you be open to attending AI training workshops on its application in hepatology research?		
Yes, definitely	166	70.3
Maybe, if the training is relevant to my work	68	28.8
No, I do not see the need	2	0.8
How should AI be integrated into hepatology training programs? *		
Hands-on workshops and AI simulation-based learning	173	73.3
AI-focused courses and certifications for hepatologists	155	65.7
Online AI learning platforms for medical professionals	145	61.4
Collaboration with AI researchers to develop hepatology-specific applications	122	51.7
Inclusion of AI education in medical school curricula	116	49.2

* Questions with multiple responses.

a Other: Validation of an AI prediction versus human for medical knowledge.

### 3.6 Qualitative data analysis

Thematic analysis of open-ended responses (Supplementary Table S1) revealed several priority areas for AI integration in hepatology across the MENA region.

The most frequently cited theme was the urgent need for comprehensive legal, ethical, and regulatory frameworks. Respondents emphasized the development of national guidelines to address accountability, data privacy, patient consent, and governance of AI use in clinical settings.

Technical challenges were also prominent. Participants highlighted the importance of standardized clinical datasets, interoperability with electronic health systems, and secure data-sharing mechanisms to ensure seamless AI integration.

Education and capacity building emerged as essential pillars, with strong support for incorporating AI training into undergraduate and postgraduate curricula, as well as offering hands-on workshops, online courses, and interdisciplinary collaboration with AI developers.

Participants supported the evidence-based inclusion of AI in clinical guidelines and stressed the need for equitable access, particularly in low-resource settings, with calls for subsidized technologies and infrastructure support. The need for robust research and clinical validation was underscored. Respondents advocated for region-specific studies to evaluate the effectiveness of AI tools across local populations and healthcare environments. Lastly, stakeholder engagement was viewed as critical. Respondents encouraged the involvement of physicians, patients, institutions, and regulatory authorities to ensure transparency, acceptance, and trust in AI-driven care. Thematic clustering yielded eight policy-relevant domains (Supplementary Table S1); representative, de-identified quotations supporting each theme are presented in Supplementary File S2.

## 4 Discussion

To the best of our knowledge, this is the first study to comprehensively assess the adoption, perceptions, and challenges of AI in hepatology across the MENA region. Our findings reveal a notable gap between enthusiasm for AI and its practical implementation. Although AI is widely recognized for its potential to transform liver disease management, particularly in diagnostics, prognostics, and decision support, its integration into routine hepatology practice remains limited. This disconnect appears largely driven by deficits in specialized training, limited infrastructural capacity, and unresolved ethical and regulatory concerns. Our sample was concentrated in Türkiye, Egypt, and Oman—consistent with the SLMENA network’s reach and the snowball design—so estimates of AI awareness, routine use, and institutional readiness likely reflect these higher-responding settings. Countries with smaller samples may differ in material ways (e.g., data-sharing rules, EHR penetration, reimbursement models, workforce training), and results should therefore be generalized to the broader MENA region with caution. Descriptively, larger-sample countries reported greater exposure to AI tools and higher institutional willingness, plausibly tracking more mature digital infrastructure and governance. Across experience strata, enthusiasm for AI’s clinical potential was broadly shared, while formal AI training remained limited in all groups. Because the study was not designed for inference, these patterns are non-inferential and should be interpreted cautiously; future work should use stratified sampling frames with country-level quotas to enhance representativeness.

Overall, most participants in our survey expressed cautious optimism about the role of AI in hepatology, particularly its potential to improve diagnosis, early disease detection, and risk assessment. While secondary applications such as administrative support and treatment planning were rated lower, they were still viewed as valuable adjuncts to clinical workflows. Several respondents anticipated improved patient outcomes and expanded access to care, although concerns persisted regarding the reliability, cost, and potential to exacerbate existing disparities in access to technology. Trust in AI remained moderate, with a strong preference for human oversight, especially in patient-facing roles. Many participants expressed hope that AI would soon become a routine part of clinical practice. These findings align with previous reports documenting a shift in physicians’ attitudes, from initial fear of human replacement to a more measured optimism regarding AI integration, albeit tempered by concerns about diminished clinical autonomy (10, 11). Notably, such concerns appear to be more strongly influenced by user characteristics than by AI performance itself (10). Bisdas et al. noted that positive attitudes were more common among individuals with prior AI exposure or those practicing in well-resourced environments, while skepticism was more prevalent among medical students and respondents from low- and middle-income countries (11). This underscores the role of training and infrastructure in shaping perceptions. Despite persistent concerns related to control, ethics, and employment, more than 60% of physicians and trainees in other studies have expressed optimism about AI (12, 13), a sentiment shared by both gastroenterologists and general practitioners (14, 15). Compared with surveys of physicians/trainees in other regions and specialties (10–15), MENA hepatologists reported similar overall optimism but lower routine use, a stronger prioritization of imaging and fibrosis staging, markedly limited formal AI training, and a pronounced preference for human oversight—patterns that are consistent with infrastructural and governance gaps described in resource-constrained settings.

Our results show that only a small proportion of participants had received formal training in AI, with nearly half reporting no prior exposure. Despite this, there was strong interest in pursuing AI education, particularly when linked to their clinical practice or research work. Respondents favored hands-on workshops and simulation-based learning as preferred formats, and frequently emphasized the importance of integrating AI into medical education and fostering collaboration with AI experts. These findings underscore the urgent need for accessible, context-specific training in AI for hepatologists.

Structured AI education for hepatology professionals remains underdeveloped, consistent with trends across other medical fields. Studies have shown that most physicians and medical trainees lack formal AI instruction despite increasing integration of AI technologies into healthcare settings. For example, a survey from United Kingdom found that 92% of trainee doctors considered their current curricula inadequate for AI training (16). Likewise, a study from Nepal highlighted limited AI knowledge among medical students and interns (17). Across multiple studies, physicians have expressed a strong desire to improve their AI literacy and called for healthcare institutions and academic bodies to implement formal training programs (10, 14, 18). In line with these observations, structured, practice-oriented curricula and modular workshops can correct misconceptions and accelerate safe adoption (19).

Previous studies have shown that medical students often rely on media and peer discussions to learn about AI, due to limited formal training in their academic curricula (20, 21). In contrast, several structured educational programs have emerged in countries such as the United States (12, 13), Canada (221), France (22), and Mexico (23). These initiatives span a variety of instructional methods, including didactic lectures (22–26), discussion-based sessions (25), web-based modules (23), workshops, case-based formats (27), and experiential learning opportunities (24). Most of these programs are implemented in academic institutions (22–26), reflecting a growing institutional commitment to AI education.

The duration and design of these programs vary considerably, ranging from single-day workshops (25) to fellowships extending beyond 1 year (24), highlighting their adaptability to diverse learner needs. This flexibility enables educators to tailor content to different time constraints, experience levels, and learning goals. Such structured approaches have been shown to correct misconceptions, increase acceptance of AI, and reinforce its role as a supportive tool rather than a threat to clinical practice (11, 28, 29).

Integrating AI into healthcare education remains challenging. Institutional barriers such as conventional teaching methods, inflexible funding models, and restrictive university policies hinder innovation. Meaningful progress will require reforms in accreditation and licensing processes to create space for dynamic, forward-thinking curricula (28, 30). Inclusion of AI competencies into medical education must also be accompanied by a multidisciplinary approach that preserves core humanistic values, particularly compassion, which remain central to high-quality patient care (31).

Despite recent advances demonstrating the utility of AI in hepatology (7), our survey indicates that its adoption in the MENA region remains limited. Approximately two-thirds of respondents believed AI’s most significant value lies in imaging analysis and liver fibrosis staging. These views are supported by emerging evidence highlighting AI’s expanding role in managing MASLD. In particular, AI-driven predictive models have been developed to estimate HCC risk in MASLD populations (32). Beyond prediction, AI is increasingly used to enhance patient stratification, discover novel biomarkers, and identify therapeutic targets by analyzing data from electronic health records, digital pathology, and imaging (33).

AI has also shown promise in diagnostic support, especially in evaluating multimodal data such as imaging and laboratory findings (34). In the context of HCC and cholangiocarcinoma, deep learning models have been applied to improve diagnostic accuracy, tumor classification, and treatment planning, as well as to predict clinical outcomes (35–37). Similarly, in liver transplantation, AI is being utilized for dynamic risk prediction, optimizing organ allocation, and forecasting post-transplant outcomes (5). Despite these advancements, our data show that AI use in transplantation among hepatologists in the MENA region remains underdeveloped. Outside the MENA region, AI has already influenced hepatology through deep-learning image analysis for lesion characterization and staging, AI-assisted elastography/fibrosis grading, and multimodal decision support that integrates imaging with laboratory and clinical data (1, 3, 5, 7, 35–37). In parallel, clinical decision support systems increasingly target risk stratification and longitudinal care pathways, aligning with domain needs in hepatology where high-dimensional data and evolving phenotypes challenge traditional tools (1, 3, 5, 7).

In the research domain, just over 40% of our respondents reported using AI tools for tasks such as literature summarization, statistical analysis, and manuscript preparation. Researchers are increasingly recognizing both the potential of AI to advance research and the challenges it presents, particularly concerns related to data bias, lack of validation, and ethical considerations (1–3). Machine learning applications now support drug discovery (38), trial optimization (39), real-time data acquisition through wearable devices (40), and improved endpoint detection and risk monitoring (41). AI also plays a role in managing missing data and enhancing participant recruitment and retention using natural language processing and passive data collection techniques (42). However, persistent challenges, including data interoperability and secure data sharing, continue to hinder the broader application of AI in clinical research.

Participants noted limited institution-wide awareness of AI across the MENA region, highlighting a persistent gap between conceptual understanding and practical readiness for implementation. While many institutions demonstrated moderate preparedness, few appeared fully committed to integrating AI into their workflows. Although AI technologies have made substantial progress in healthcare, institutional readiness to adopt these innovations remains highly variable, hindered by multiple challenges and unresolved operational, infrastructural, and regulatory considerations (43). Respondents identified several key barriers to AI adoption in hepatology across the MENA region, including limited expertise, restricted access to AI tools, inadequate infrastructure, and unresolved regulatory issues. Ethical concerns were also prevalent, particularly regarding data security, algorithmic bias, and potential over-reliance on AI systems. These concerns align with findings from the broader international literature, which frequently cites the absence of ethical and legal standards as major impediments to AI integration in healthcare (15, 44, 45). To address these challenges, participants emphasized the need for clear regulatory frameworks, targeted training programs, rigorous clinical validation, and transparent collaboration between stakeholders. Data security and privacy risks have also been widely acknowledged (46). In addition, the lack of explainability, often referred to as the “black box” nature of AI, remains a challenge, as clinicians may struggle to interpret or trust AI-generated outputs (47). Another critical issue is the underrepresentation of minority and rural populations in AI training datasets, which undermines the generalizability and equity of AI models (48). Allocation of ethical and legal responsibility for AI-supported decisions remains jurisdiction-dependent and evolving. Existing guidance emphasizes that developers are accountable for design quality, data provenance, transparency, and post-deployment monitoring, whereas implementers and institutions are responsible for validation in-context, governance, and safe integration into clinical workflows; clinicians retain ultimate accountability for patient-facing decisions (4, 6, 48, 49). In the MENA region—where regulatory maturity and data-sharing rules vary—pragmatic “human-in-the-loop” deployment, institutional oversight, and clear audit trails are likely to be essential transitional safeguards until harmonized medico-legal frameworks are established. The concerns voiced by MENA hepatologists—privacy/confidentiality, bias and reliability, explainability, liability, and the necessity of human oversight—mirror priorities in international guidance (e.g., WHO recommendations) and EU-level initiatives emphasizing transparency, accountability, safety, and context-specific validation (4, 48, 50). Our results therefore reinforce the importance of adapting these global principles to local legal environments, data-governance capacity, and workflow realities.

In line with surveys from North America, Europe, and Asia, clinicians in our cohort reported high interest but tempered trust, with persistent concerns about privacy, liability, explainability, and cost (10–15, 18, 20, 44–47). Common barriers mirrored international reports, limited formal training, lack of integration with EHR/EMR systems, and unclear governance (10–15, 18, 20, 44–47). Relative to many high-income settings, however, MENA hepatologists reported lower routine use, more limited formal AI training, and stronger emphasis on infrastructure and regulatory readiness, consistent with challenges described in LMIC contexts (49). Notably, our respondents prioritized imaging and fibrosis staging more than some non-hepatology specialties, aligning with domain-specific opportunities in hepatology (1, 3, 5, 7, 35–37). These contrasts suggest that implementation strategies in the region should pair education with investments in interoperability, standards, and governance.

Participants emphasized the importance of establishing robust ethical and legal frameworks, ensuring data privacy, and achieving interoperability between AI systems and existing healthcare infrastructure. There was broad consensus on the need to integrate AI education into medical curricula to foster collaboration between clinicians and technology developers. While AI holds significant promise, especially in diagnostics and workflow optimization, participants stressed that it must complement, rather than replace, clinical judgment and the human aspect of care. Equitable access, affordability, and inclusive stakeholder engagement were identified as foundational to the responsible and sustainable implementation of AI in hepatology. These findings align with reports exploring regional perspectives on AI deployment in healthcare systems. Persistent technical issues such as algorithmic bias, overfitting, and limited generalizability are increasingly being addressed through region-specific datasets, stakeholder involvement, and rigorous validation protocols (4, 51). Evidence suggests that AI models trained on locally relevant data perform better and are more applicable to real-world practice (4, 51). In low- and middle-income countries (LMICs), leveraging locally sourced data, mobile health (mHealth) tools, and cloud-based infrastructures can help overcome limitations related to data quality and system capacity (49). Explainable AI is also gaining attention, enabling clinicians to better understand algorithmic outputs and enhancing trust (4). Ethical and legal challenges are being met through anonymization techniques, regulatory reforms, and international policy harmonization (49, 50). A notable example of global collaboration’s impact is a Google-led multicenter initiative that reduced false-positive and false-negative rates by 5–7%, thereby improving diagnostic accuracy and illustrating the value of targeted training and cross-sector partnerships (4). Region-aware governance and proactive stakeholder alignment further improve feasibility and uptake (52). Beyond diagnosis and staging, machine-learning models increasingly aim to forecast decompensation, mortality, and transplant-free survival, and to complement or recalibrate conventional scores (e.g., MELD) by leveraging non-linear interactions across clinical, imaging, and biomarker data. Early studies in ACLF and transplant pathways suggest potential for earlier risk identification, dynamic prioritization, and improved post-transplant outcome prediction; however, prospective validation and equity checks are essential before routine use (53, 54).

This study presents several notable strengths. It is the first comprehensive assessment of AI integration into hepatology practice and research across the MENA region, offering valuable regional insight where limited data currently exist. The study achieved a high response rate of 82.8%, with representation from 17 countries, enhancing its generalizability across diverse clinical settings. The use of a mixed-methods approach, combining quantitative data with thematic analysis of open-ended responses, allowed for an in-depth understanding of hepatologists’ perceptions, challenges, and recommendations. The survey instrument underwent pilot testing to ensure clarity and content validity, and the discussion is well-grounded in global literature, providing a contextual interpretation of the findings. These findings offer a foundation for future longitudinal and interventional research, emphasizing the urgent need for structured training, regulatory guidance, and strategic investment to facilitate the responsible integration of AI into hepatology practice.

This study has some limitations. First, reliance on self-reported perceptions may introduce recall, perception, and social-desirability bias; although anonymity and voluntary participation were used to mitigate this, reported use and trust may be over- or underestimated. Second, the cross-sectional design precludes causal inference and assessment of temporal trends; longitudinal follow-up is warranted to track changes in adoption and readiness. Third, the non-probability convenience/snowball recruitment may have introduced selection bias toward clinicians with stronger views about AI, limiting generalizability beyond our sample. Fourth, country-level numbers were small for several settings; accordingly, between-country comparisons are descriptive only and should be interpreted cautiously. Fifth, institutional readiness and AI use were not independently verified against records, and we did not collect objective clinical outcomes or detailed performance characteristics of specific AI tools. Given the sampling design and sparse strata, we prespecified a descriptive analytic approach and did not perform inferential hypothesis testing to avoid overstating population-level inference. Finally, missing data were handled by case-wise omission and some items permitted multiple responses; thus, denominators vary and percentages may not sum to 100%. Despite these constraints, the study’s multinational scope, high response rate, and mixed-methods design provide foundational, region-specific insights to guide policy, training, and implementation strategies for AI in hepatology.

## 5 Conclusion

AI adoption in MENA hepatology is characterized by high interest but limited routine use. Priority actions include (i) region-appropriate governance with clear accountability and human-in-the-loop safeguards; (ii) data standards and interoperability to enable integration with EHR/EMR and imaging systems; (iii) scalable training pathways—curricular integration, workshops, and simulation; and (iv) equitable access supported by local validation on MENA datasets. Coordinated efforts by ministries, professional societies, and academic centers are essential to translate optimism into safe, routine clinical benefit.

## Data Availability

The original contributions presented in the study are included in the article/Supplementary material, further inquiries can be directed to the corresponding author.

## Group member of Steatotic Liver Disease Study Foundation in Middle East and North Africa (SLMENA) Collaborators

Heba Shafeak Abd El Khalik, Internal Medicine, Zagazig University, Zagazig, Egypt; Summer Saad Abdalhussain, Medicine, Kirkuk Medical College, Kirkuk, Iraq; Yasser Abdelghani, Department of Gastroenterology, Hepatology and Endemic Medicine, Minia University, Minia, Egypt; Mohamed Omar Abdelmalek, Tropical Medicine and Gastroenterology, Assiut University, Assiut, Egypt; Aya Milod Abdo, Gastroenterology, Tripoli Central Hospital, Tripoli, Libya; Ali Adil Abdulhussein, Medicine, Kirkuk Medical College, Kirkuk, Iraq; Maheeba Abdulla, Internal Medicine, Ibn AlNafees Hospital, Manama, Bahrain; Aras Aziz Abdulla, Gastroenterology & Hepatology Subspecialty Program, Kurdistan Higher Council of Medical Specialties (KHCMS), Sulaimani, Iraq; Harem Sattar Abdulqader, Medicine, Kirkuk Medical College, Kirkuk, Iraq; Hossameldin Fathy Abou Elkhier, Tropical Department, Alexandria University, Alexandria, Egypt; Ayman Mohammed AbuHelal, Rheumatology Department, Jordan University Hospital, Amman, Jordan; Imad Mohammad Abumallouh, Medical Department, Armed Force Hospital Alkhoud, Muscat, Oman; Gupse Adali, Gastroenterology, University of Health Sciences Istanbul Umraniye Training and Research Hospital, Istanbul, Türkiye; Abdalaziz Omar Adali, Gastroenterology, Tripoli Central Hospital, Tripoli, Libya; Ahmed Hatim Moahmed Ahmed, General Medicine, Hamad Medical Corporation, Doha, Qatar; Hasnaa Mohammed Ahmed, Department of Gastroenterology, Hepatology and Endemic Medicine, Minia University, Minia, Egypt; YASIR Khudhair Ahmed, College of Medicine, Tikrit University, Tikrit, Iraq; Mohammed Hussien Ahmed Hussien, Gastroenterology, Hepatology and Infectious Disease, Kafrelsheikh University, Kafrelsheikh, Egypt; Halit Tugrul Akarca, Gastroenterology, Ankara University Faculty of Medicine, Ankara, Türkiye; Mehmet Akca, Gastroenterology, Ondokuz Mayıs University, Samsun, Türkiye; Remzi A. Akdogan, Gastroenterology, Recep Tayyip Erdogan University, Rize, Türkiye; Meral Akdogan Kayhan, Department of Gastroenterology, Health Science University, Bilkent City Hospital, Ankara, Türkiye; Murat Akyildiz, Gastroenterology and Hepatology, Koç University School of Medicine, Istanbul, Türkiye; Seyma Handan Akyon, Home Care Services, Sincan Education and Research Hospital, Ankara, Türkiye; Said Al Alawi, Gastroenterology, Sur Hospital, Sur, Oman; Kawkab Salim Al Balushi, Medical City for Military and Security Services, Muscat, Oman; Nooreen Yousuf Al Balushi, Royal Hospital, Muscat, Oman; Yousuf Said Mahmood AL Balushi, Medical City for Military and Security Services, Muscat, Oman; Badriya S Al Farsi, Medical City for Military and Security Services, Muscat, Oman; Fat’hiya Mohammed Al Harthy, Royal Hospital, Muscat, Oman; Al Warith Al Hashmi, Royal Hospital, Muscat, Oman; Salah Al Jabri, Royal Hospital, Muscat, Oman, Hour Al Kaabi, Medical City for Military and Security Services, Muscat, Oman; Maimouna N Al Mandhari, Royal Hospital, Muscat, Oman; Nasser M AL Masri, Gastroenterology & Hepatology, Prince Sultan Medical Military, Riyadh, Saudi Arabia; Jawahar H Al Noumani, Sultan Qaboos University Hospital, Muscat, Oman; Issa Saif Al Qarshoubi, Gastroenterology & Advanced Endoscopy, Sultan Qaboos Comprehensive Cancer Care & Research Centre, Muscat, Oman; Halima Hamed Al Shuaili, Medical City for Military and Security Services, Muscat, Oman; Siham Al Sinani, Sultan Qaboos University Hospital, Muscat, Oman; Motasam Belah Al Swayah, Gastroenterology, Tripoli Central Hospital, Tripoli, Libya; Abdulnasser Aladawi, Medical City for Military and Security Services, Muscat, Oman; Ali Alali, Medicine, Kuwait University, Kuwait City, Kuwait; Abduljaleel Alalwan, Hepatobiliary and Liver Transplant Unit, King Abdulaziz Medical City, Riyadh, Saudi Arabia; AL-Qasim AL-Bahlani, Royal Hospital, Muscat, Oman; Fatih Albayrak, Gastroenterology, Ataturk University, Erzurum, Türkiye; Mustafa M. Aldaqrum, Gastroenterology, Tripoli Central Hospital, Tripoli, Libya; Ahmed K Al-Darmaki, Royal Hospital, Muscat, Oman; Hisham Abdullah AlDhahab, Royal Hospital, Muscat, Oman; Saad Aldosari, Medicine, King Abdulaziz Medical City, Jeddah, Saudi Arabia; Samer Al-Dury, Medical City for Military and Security Services, Muscat, Oman; Ahmed Abdurrahman Algeblawi, Gastroenterology, Tripoli Central Hospital, Tripoli, Libya; Abdullah Saeed Alghamdi, Medical/Gastroenterology Unit, King Fahad Hospital, Jeddah, Saudi Arabia; Mohammed Y Alghamdi, Medicine, King Fahd Military Medical Complex, Dhahran, Saudi Arabia; Nadya Abdullah AlHarthy, Royal Hospital, Muscat, Oman; Omar A Alhusayni, Medicine, King Abdulaziz Medical City, Jeddah, Saudi Arabia; Randa S Ali, Department of Gastroenterology, Hepatology and Endemic Medicine, Minia University, Minia, Egypt; Wesam Saif Ali, Hepatology and Gastroenterology, NHTMRI, Cairo, Egypt; Mohammed Aljawad, Medicine, Johns Hopkins Aramco Healthcare, Dhahran, Saudi Arabia; Abdullah Humaid Alkalbani, Medical City for Military and Security Services, Muscat, Oman; Nawal Mehdi Alkhalidi, Medicine, Git Teaching Hospital, Baghdad, Iraq; Fahd Almalki, Medicine, Umm Al-Qura University, Makkah, Saudi Arabia; Mohammad Jameel Almatrafi, National Guard Hospital-Gastroenterology Department, Al Madinah Al-Munawarah, Saudi Arabia; Zakariya AL-Naamani, Sultan Qaboos University Hospital, Muscat, Oman; Faisal Mania Alotaibi, Adult Gastroenterology and Hepatology Department, King Fahad Medical City, Riyadh, Saudi Arabia; Hassan Abdullah Alqartoobi, Medical City for Military and Security Services, Muscat, Oman; Farooq Fouad Alrawi, General Medicine, Hamad Medical Corporation, Doha, Qatar; Salim Mohammed Alriyami, Royal Hospital, Muscat, Oman; Abdulmalik M Alsabban, Gastroenterology, Prince Mohammad Bin Abdulaziz Hospital, Medina, Saudi Arabia; Marwan T A Alsari, Gastroenterology, Tripoli Central Hospital, Tripoli, Libya; Mohamed A Alsenbesy, Internal Medicine, Ibn AlNafees Hospital, Manama, Bahrain; Omar Alsiyabi, Royal Hospital, Muscat, Oman; Abdulatif Alsuleimani, Medical City for Military and Security Services, Muscat, Oman; Jamal Abdulnasser Altamimi, Gastroenterology, Ibn Sina General Hospital, Hadrmoot, Yemen; Loai A Altayib, Gastroenterology, National Medical Care, Riyadh, Saudi Arabia; Adnan Alzanbagi, Gastroenterology, King Abdullah Medical City, Makkah, Saudi Arabia; Derya Arı, Gastroenterology, Ankara Bilkent City Hospital, Ankara, Türkiye; İpek Bilge Aslan, Gastroenterology, Ege University, Izmir, Türkiye; Ahmed Mohamed Assar, Hepatology and Gastroenterology, NHTMRI, Cairo, Egypt; Mohamed L. Asser, Hepatology and Gastroenterology, Medical Research Institute, Alexandria, Egypt; Halil Atasoy, Gastroenterology, Recep Tayyip Erdogan University, Rize, Türkiye; Dina Attia, Gastroenterology, Hepatology & Endemic Medicine, Beni-Suef University, Beni Suef, Egypt; Gokhan Aydin, Department of Gastroenterology, Giresun University Faculty of Medicine, Giresun, Türkiye; Rifat Furkan Aydın, Internal Medicine, Ankara University, Ankara, Türkiye; Mohammed Diaaldin Ayoub, Pediatrics, King Abdulaziz University, Jeddah, Saudi Arabia; Soran Baban, Gastroenterology & Hepatology Subspecialty Program, Kurdistan Higher Council of Medical Specialties (KHCMS), Sulaimani, Iraq; Yasemin Hatice Balaban, Gastroenterology, Hacettepe University, Ankara, Türkiye; Metin Basaranoglu, Gastroenterology, Bezmialem University, Istanbul, Türkiye; Dalia Basulayman, Medicine, King Abdulaziz Medical City, Jeddah, Saudi Arabia; Reem Abdulhay Ben Omran, Gastroenterology, Tripoli Central Hospital, Tripoli, Libya; Camélia Berhili, Liberal Sector, Rabat, Morocco; Ruchir Kumar Bichu, Medical City for Military and Security Services, Muscat, Oman; Mehmet Biricik, Internal Medicine, Kızıltepe State Hospital, Mardin, Türkiye; Ali Rıza Çalışkan, Department of Gastroenterology, Adıyaman University, Adıyaman, Türkiye; Rasim E. Cankurtaran, Gastroenterology, Ankara Yıldırım Beyazıt University, Ankara, Türkiye; Deha Duran Cetin, Gastroenterology, Adana City Hospital, Adana, Türkiye; Sedat Çiçek, Gastroenterology, Firat University, Elazığ, Türkiye; Hatice Colak, Nutrition and Dietetics, Istanbul Bilgi University, Istanbul, Türkiye; Arif Mansur Cosar, Gastroenterology, Karadeniz Technical University, Trabzon, Türkiye; Nabil Debzi, Hepatology, Mustapha Bacha University Hospital, Algiers, Algeria; Hakan Demiröz, Gastroenterology, Ondokuz Mayıs University, Samsun, Türkiye; Berkay Dertsiz, Gastroenterology, Ümraniye Training and Research Hospital, Istanbul, Türkiye; Elham Salah Desokey, Tropical Medicine and Gastroenterology, Aswan University, Aswan, Egypt; Hakan Dursun, Gastroenterology, Ataturk University, Erzurum, Türkiye; Maissa E El Raziky, Endemic Medicine, Cairo University, Cairo, Egypt; Reda Mohamed Elbadawy, Gastroenterology, Hepatology, Benha University, Benha, Egypt; Nada El-Domiaty, Endemic Medicine Department, Helwan University, Cairo, Egypt; Asem Elfert, Tropical Medicine, Tanta University Faculty of Medicine, Tanta, Egypt; Ahmed Belgasem Elhaddad, Gastroenterology, Tripoli Central Hospital, Tripoli, Libya; Ahmed F Elhassi, Gastroenterology, Tripoli Central Hospital, Tripoli, Libya; Hanaa Mostafa El-Karaksy, Endemic Medicine, Cairo University, Cairo, Egypt; Mumajed Lutfi Elmsellati, Gastroenterology, Tripoli Central Hospital, Tripoli, Libya; Mohamed Hassan Elnadry, Hepatology, Gastroenterology & Infectious Diseases, Al-Azhar University, Cairo, Egypt; Fatma Elrashdy, Endemic Medicine, Cairo University, Cairo, Egypt; Medhat Elsahhar, Gastroenterology and Hepatology, Police Authority Hospitals, Cairo, Egypt; Mortada H. F. El-Shabrawi, Endemic Medicine, Cairo University, Cairo, Egypt; Eman E. Elshemy, Hepatology, Gastroenterology & Infectious Diseases, Al-Azhar University, Cairo, Egypt; Iman Elsherif, Department of Internal Medicine, Suez Canal University, Ismailia, Egypt; Doaa Elwazzan, Tropical Department, Alexandria University, Alexandria, Egypt; Ghada Younes Elzewiei, Gastroenterology, Tripoli Central Hospital, Tripoli, Libya; Ilkay Ergenç, Hepatology, King's College Hospital, London, UK; Murat Erkut, Gastroenterology, Karadeniz Technical University, Trabzon, Türkiye; Hasan Eruzun, Gastroenterology, Ondokuz Mayıs University, Samsun, Türkiye; Mohammed Ezz-Eldin, Tropical Medicine and Gastroenterology, Assiut University, Assiut, Egypt; Mohanad Faisal, General Medicine, Hamad Medical Corporation, Doha, Qatar; Eman M. Fares, Endemic Medicine, Fayoum University, Fayoum, Egypt; Sami Fidan, Gastroenterology, Karadeniz Technical University, Trabzon, Türkiye; Mahrez Fissah, Hepatology, Mustapha Bacha University Hospital, Algiers, Algeria; Mostafa Taha Gabr, Tropical Medicine, Tanta University Faculty of Medicine, Tanta, Egypt; Ghada M Galal, Tropical Medicine and Gastroenterology, Assiut University, Assiut, Egypt; Saleh A. Garad, Gastroenterology & Hepatology, Al-gamhuria Modern General Hospital, Aden, Yemen; Prof A. A. Gehani, General Medicine, Hamad Medical Corporation, Doha, Qatar; Ahmed Gouda, Gastroenterology, Burjeel Medical Centre, Abu Dhabi, UA; Donia Gouiaa, Gastroenterology, La Rabta Hospital, Tunis, Tunisia; Gamze Gül, Nutrition and Dietetics, Izmir Democracy University, Izmir, Türkiye; Murat Taner Gülşen, Gastroenterology, Gaziantep University, Gaziantep, Türkiye; Burcu Gurbuz, Gastroenterology, Hacettepe University, Ankara, Türkiye; Khaled Mohd Hamad, Medical City for Military and Security Services, Muscat, Oman; Aya Hammami, Department of Gastroenterology, University Hospital of Sahloul, Sousse, Tunisia; Mustafa Harı, Gastroenterology, Adana City Hospital, Adana, Türkiye; Zekiye Nur Harput, Gastroenterology, Tarsus State Hospital, Mersin, Türkiye; Dalia Hassan, Internal Medicine, Petroleum Hospital, Alexandria, Egypt; Norhan Ali Hassan, Department of Gastroenterology, Hepatology and Endemic Medicine, Minia University, Minia, Egypt; Marwa Khalaf Hassan, Tropical Medicine and Gastroenterology, Assiut University, Assiut, Egypt; Gdoura Hela, Gastroenterology, La Rabta Hospital, Tunis, Tunisia; Amal A Helmy, Endemic Medicine, Cairo University, Cairo, Egypt; Sayed Mohamed Hemdan, Medicine, Kuwait University, Kuwait City, Kuwait; Abdullah İlhan, Gastroenterology, Adana City Hospital, Adana, Türkiye; Mona Ismail, Division of Gastroenterology, Imam Abdulrahman bin Faisal University, Alkhobar, Saudi Arabia; Kemal Yunus İyiyapıcı, Gastroenterology, Gaziantep University, Gaziantep, Türkiye; Amar Jasem, Medicine, Kuwait University, Kuwait City, Kuwait; Bola Rajendra Kamath, Medical City for Military and Security Services, Muscat, Oman; Saibe Merve Kazdal, Gastroenterology, Recep Tayyip Erdogan University, Rize, Türkiye; Caglayan Keklikkiran, Gastroenterology, Recep Tayyip Erdogan University, Rize, Türkiye; Emre Batuhan Kenger, Nutrition and Dietetics, Istanbul Bilgi University, Istanbul, Türkiye; Onur Keskin, Gastroenterology, Hacettepe University, Ankara, Türkiye; Yusuf Bünyamin Ketenci, Gastroenterology, Ondokuz Mayıs University, Samsun, Türkiye; Nahla Ahmed Khalaf, Tropical Medicine, Tanta University Faculty of Medicine, Tanta, Egypt; Mohammed Adnan Khan, Medical City for Military and Security Services, Muscat, Oman; Fatih Kıvrakoğlu, Gastroenterology, Adana City Hospital, Adana, Türkiye; Ayşenur Kördüğüm, Gastroenterology, Gaziantep University, Gaziantep, Türkiye; Hüseyin Köseoğlu, Gastroenterology, Hitit University, Çorum, Türkiye; Norhan Osama Mady, Department of Gastroenterology, Hepatology and Endemic Medicine, Minia University, Minia, Egypt; Reem Ezzat Mahdy, Tropical Medicine and Gastroenterology, Assiut University, Assiut, Egypt; Nahed A Makhlouf, Tropical Medicine and Gastroenterology, Assiut University, Assiut, Egypt; Khaled Matar, Gastroenterology Department, European Gaza Hospital, Gaza Strip, Palestine; Rasha Matar, Gastroenterology & Advanced Endoscopy, Sultan Qaboos Comprehensive Cancer Care & Research Centre, Muscat, Oman; Mohammad Mawardi, Medicine, King Abdulaziz Medical City, Jeddah, Saudi Arabia; Sahide N. I. Melez, Gastroenterology, Gaziantep University, Gaziantep, Türkiye; Aymen Nassem Menessy, Hepatology & Gastroenterology Unit, Mansoura Faculty of Medicine, Mansoura, Egypt; Bilge Meral Koç, Nutrition and Dietetics, Izmir Democracy University, Izmir, Türkiye; Adnan Misellati, General Medicine, Hamad Medical Corporation, Doha, Qatar; Taha Ahmad Mohamad, Gastroenterology & Hepatology Subspecialty Program, Kurdistan Higher Council of Medical Specialties (KHCMS), Sulaimani, Iraq; Abrar Mohamed, Department of Gastroenterology, Hepatology and Endemic Medicine, Minia University, Minia, Egypt; Omed Oubed Mohammad, Rizgary Teaching Hospital, Erbil, Iraq; Malavan H. Mohammed, Department of Internal Medicine, University of Duhok, Duhok, Iraq; Khairy Hammam Morsy, Tropical Medicine and Gastroenterology, Sohag University, Sohag, Egypt; Hussein M Mossa, Department of Gastroenterology, Hepatology and Endemic Medicine, Minia University, Minia, Egypt; Ehab F Moustafa, Tropical Medicine and Gastroenterology, Assiut University, Assiut, Egypt; Benazzouz Mustapha, Liberal Sector, Rabat, Morocco; Hanaa Mahmoud Nagdy, Internal Medicine, Arab Academy for Science Technology and Maritime Transport, Alexandria, Egypt; Youssef Eshak Naguib, Hepatology and Gastroenterology, NHTMRI, Cairo, Egypt; Husna Omran, Medical City for Military and Security Services, Muscat, Oman; Youssef Hadi Osman, Gastroenterology, Tripoli Central Hospital, Tripoli, Libya; Murat Ozdede, Gastroenterology, Hacettepe University, Ankara, Türkiye; Tugce Ozlu Karahan, Nutrition and Dietetics, Istanbul Bilgi University, Istanbul, Türkiye; Bengi Öztürk, Gastroenterology, Hacettepe University, Ankara, Türkiye; Yavuz E Parlar, Gastroenterology, Ankara Training and Research Hospital, Ankara, Türkiye; Yunus Halil Polat, Gastroenterology, Ankara Training and Research Hospital, Ankara, Türkiye; Almotasembillah Rammal, Medicine, King Abdulaziz Medical City, Jeddah, Saudi Arabia; Dr. Asia Salah Rasool, Gastroenterology & Hepatology Subspecialty Program, Kurdistan Higher Council of Medical Specialties (KHCMS), Sulaimani, Iraq; Mehmet Ali Şahan, Gastroenterology, Gaziantep University, Gaziantep, Türkiye; Yara Mohamed Salah, Department of Gastroenterology, Hepatology and Endemic Medicine, Minia University, Minia, Egypt; Nairoz Alnuri Sasi, Gastroenterology, Tripoli Central Hospital, Tripoli, Libya; Serhat Saylam, Internal Medicine, Akcakoca Government Hospital, Duzce, Türkiye; Ebubekir Şenateş, Gastroenterology, Acıbadem Taksim Hospital, Istanbul, Türkiye; Hande Seven Avuk, Nutrition and Dietetics, Istanbul Bilgi University, Istanbul, Türkiye; Mohammed Raziuddin Shajee, Medical City for Military and Security Services, Muscat, Oman; Mona Helmy Shehata, Tropical Medicine, Tanta University Faculty of Medicine, Tanta, Egypt; Hend I Shousha, Endemic Medicine, Cairo University, Cairo, Egypt; İbrahim Halil Söylemez, Gastroenterology, Gaziantep University, Gaziantep, Türkiye; Cemal Firat Subasi, Gastroenterology and Hepatology, Istanbul Gaziosmanpaşa Training and Research Hospital, Istanbul, Türkiye; Marlain Odeh Swaiss, Trizara Nutrition Center, Amman, Jordan; Said Taharboucht, Hepatology, Mustapha Bacha University Hospital, Algiers, Algeria; Mostafa Tharwat, Internal Medicine, Zagazig University, Zagazig, Egypt; Melike Tuncer, Dietitian, Koç University, Istanbul, Türkiye; Ilker Turan, Gastroenterology, Ege University, Izmir, Türkiye; Dilara Turan Gökçe, Gastroenterology, Ankara Training and Research Hospital, Ankara, Türkiye; Gaye Ulgut, Gastroenterology, Ege University, Izmir, Türkiye; Yilmaz Usluoglu, Gastroenterology, Ege University, Izmir, Türkiye; Muge Ustaoglu, Gastroenterology, Ondokuz Mayıs University, Samsun, Türkiye; Imam Waked, Hepatology, National Liver Institute, Shebeen ElKom, Egypt; Marwa Y, Medical City for Military and Security Services, Muscat, Oman; Haythem Yacoub, Gastroenterology, La Rabta Hospital, Tunis, Tunisia; Cesar Yaghi Hepatology and Gastroenterology, Saint Joseph University of Beirut, Lebanon; Alper Yurci, Gastroenterology, Medicana Ataköy, Istanbul, Türkiye; Moshera Ahmed Zaki, Gastroenterology, New Cairo Hospital, Cairo, Egypt; Ysear Zakri, Gastroenterology, Hepatology and Endoscopy, Prince Sultan Military Medical City, Riyadh, Saudi Arabia.
