# Supplementary material for: Mapping artificial intelligence adoption in hepatology practice and research: challenges and opportunities in MENA region
Source: Front Med (Lausanne). 2025 Sep 12;12:1630831. doi: 10.3389/fmed.2025.1630831 (PMC12463891; doi:10.3389/fmed.2025.1630831)
Supplement: Supplementary file 1 [file Supplementary_file_1.docx]

**^Survey on Hepatologists’ Perceptions of Artificial Intelligence in Liver Diseases in the MENA Region^**

**^Introduction^**

^Artificial Intelligence (AI) is increasingly being applied in hepatology for diagnosis, treatment planning, and patient management. This survey aims to assess the awareness, utilization, perceptions, and barriers to AI adoption among hepatologists in the MENA region. Your valuable input on national policies, guidelines, diagnostic practices, treatment approaches, resource availability, and the integration of AI in hepatology will help shape future strategies for AI implementation.^

^The data gathered will identify key strengths and challenges in current AI applications and inform evidence-based recommendations for improving AI integration in hepatology across the MENA region.^ **^This survey will take approximately 10 minutes to complete. Your participation in this study will be acknowledged in the final publication.^**

^This study has received ethical approval from the Research Ethics Committee at the Faculty of Medicine, Helwan University, Egypt. All responses will be kept strictly confidential and used solely for research purposes. Your participation is voluntary, and you may exit the survey at any time. Thank you for your time and contributions to this vital research.^

**^Do you wish to participate in this survey?^**

^Yes, let's start^

^No, exit^

**^Section 1: Demographics and Professional Information^**

1. **^Please provide your full name^** ^(optional, for publication purposes if desired)^

*^Example: Ahmed M. Ali^*

- ^First name: ________^
- ^Middle name initial (if applicable): ________^
- ^Last name: ________^

1. **^Please provide your affiliation^** ^(for publication purposes if desired)^*^Example: Department of Endemic Medicine, Helwan University, Cairo, Egypt^*

- ^Department Name: ________^
- ^Institution Name: ________^
- ^City: ________^
- ^Country: ________^

1. **^Please provide your email^** ^(optional, for publication purposes if desired)^
2. **^Gender:^**
   - ^Woman^
   - ^Man^
   - ^Prefer not to say^
3. **^Age Group (years):^**
   - ^25-35^
   - ^36-45^
   - ^46-55^
   - ^56-65^
   - ^>65^
4. **^Primary Specialty:^**
   - ^Hepatology^
   - ^Gastroenterology^
   - ^Internal Medicine^
   - ^Transplant Hepatology^
   - ^Other (please specify)^
5. **^Country where you currently work^** ^(Dropdown list of MENA countries)^
6. **^What has been your primary sector of work?^**
   - ^Academia (University/Teaching Hospital)^
   - ^Public Hospital^
   - ^Private Practice^
   - ^Research Institution^
   - ^Civil Society/NGO^
   - ^Other (please specify)^
7. **^What has been your primary field of work?^**

- ^Clinician/Medical Doctor^
- ^Healthcare Administration^
- ^Clinical Research^
- ^Non-Clinical Research (Epidemiology, Health Services Research, etc.)^
- ^Patient Advocacy^
- ^Policy and Health Regulation^
- ^Education/Pedagogy^
- ^Other (please specify)^

1. **^Number of peer-reviewed publications on hepatology:^**

- ^1-5^
- ^6-25^
- ^26-50^
- ^51-100^
- ^>100^

1. **^Years of experience managing patients with liver diseases:^**

- ^<5 years^
- ^5-10 years^
- ^11-20 years^
- ^>20 years^

**^Section 2: AI Awareness and Familiarity in Hepatology^**

1. **^Have you received any formal training in AI applications in medicine?^**

- ^Yes, I have completed AI-related training courses^
- ^No, but I have attended AI-related workshops or conferences^
- ^No, but I have independently studied relevant AI concepts^
- ^No, I have no prior AI training^
- ^Other (please specify)^

1. **^How frequently do you use AI-based tools in your clinical hepatology practice?^**

- ^Daily^
- ^Weekly^
- ^Monthly^
- ^Rarely^
- ^Never^

1. **^Which of the following AI-powered tools have you used in your hepatology clinical practice? (^**^Select all that apply)^

- ^AI-driven imaging analysis (e.g., fibrosis staging, AI-assisted ultrasound, MRI, CT)^
- ^AI-enhanced liver biopsy and histopathology interpretation^
- ^AI-powered prediction models for MASLD/MASH progression and HCC risk^
- ^AI-driven decision support for liver transplantation eligibility^
- ^AI chatbots or virtual assistants for patient education and engagement^
- ^Other (please specify)^
- ^I have not used AI tools in my practice^

**^Section 3: AI Perception, Utility, and Future Adoption^**

1. **^To what extent do you believe AI can enhance hepatology practice?^** ^[Likert Scale]^

- ^1 (No enhancement) to 10 (Highly transformative)^
- **^Which areas of hepatology do you think AI can contribute to most?^** ^(Rank top three in order of importance)^
- ^Diagnosis and early detection of liver diseases^
- ^Risk stratification and prognosis prediction^
- ^Treatment planning and decision support^
- ^Automating administrative tasks (e.g., patient scheduling, documentation)^
- ^Enhancing patient education and adherence^

1. **^What is your biggest concern regarding AI integration in hepatology?^** ^(Rank in order of importance)^

- ^Accuracy and reliability of AI-driven diagnostics^
- ^Data privacy and ethical concerns^
- ^Dependence on AI leading to reduced clinical skills^
- ^Cost and financial barriers^
- ^Regulatory and legal issues^
- ^Other (please specify)^

1. **^How soon do you think AI will become a routine part of hepatology practice in your institute?^**

- ^Already widely used^
- ^Within 1-3 years^
- ^Within 4-7 years^
- ^More than 7 years^
- ^Uncertain^

1. **^In your opinion, What policy changes are needed for AI adoption in hepatology?^** ^(Open-ended)^

**^Section 4: AI Implementation Challenges in Hepatology^**

1. **^What are the biggest barriers to AI adoption in hepatology practice in your institute ?^** ^(Select all that apply)^

- ^High cost of AI technology and infrastructure^
- ^Limited access to AI-driven diagnostic tools and software^
- ^Lack of technical expertise and specialized training for hepatologists^
- ^Resistance to change among healthcare professionals^
- ^Lack of standardized regulatory frameworks for AI implementation in healthcare^
- ^Ethical concerns regarding AI use in medical decision-making^
- ^Insufficient AI infrastructure in hospitals and research institutions^
- ^Lack of adaptability to tailor to patient needs^
- ^Poor responsiveness of AI models to new and emerging data and paradigms^
- ^Lack of integration with electronic medical records (EMRs) and hospital systems^
- ^Limited collaboration between AI developers and hepatologists^
- ^Other (please specify)^

1. **^How would you rate the willingness of your institution to implement AI in hepatology?^** ^[Likert Scale]^

- ^1 (Not willing at all) to 10 (Very willing)^

1. **^How do you think AI implementation should be prioritized in hepatology clinical practice ?^** ^(Rank the following based on importance)^

- ^AI-driven imaging analysis and liver fibrosis staging (e.g., AI-assisted ultrasound, MRI, CT)^
- ^AI-assisted liver transplantation evaluation and patient selection^
- ^Predictive modeling for MASLD progression and disease severity^
- ^Automated interpretation of histopathology and liver biopsy results^
- ^AI-driven treatment decision-making support for hepatologists^
- ^AI-powered chatbots and virtual assistants for patient counseling^

**^Section 5: AI and Patient Outcomes^**

1. **^How do you perceive the impact of AI on patient outcomes in hepatology?^**

- ^Negative impact - AI may increase errors or misdiagnosis^
- ^Somewhat negative impact - AI may not be reliable in clinical settings^
- ^Neutral - AI impact is uncertain^
- ^Somewhat positive impact - AI may assist in some areas but is not essential^
- ^Highly positive impact - AI will significantly improve hepatology practice^

1. **^In your opinion, will AI improve access to hepatology care in underserved areas?^**

- ^Yes, significantly – AI can bridge the gap in resource-limited settings^
- ^Somewhat – AI can assist but may not fully replace hepatologists^
- ^No impact – AI will not change accessibility issues^
- ^May worsen disparities – AI access may be limited to wealthier regions^

1. **^Do you think AI will contribute to reducing healthcare costs in hepatology?^**

- ^Yes, AI can streamline workflows and reduce unnecessary procedures^
- ^No, AI implementation may introduce additional costs^
- ^Uncertain, AI's cost-effectiveness depends on the healthcare system^

**^Section 6: Ethical Considerations and Trust in AI^**

1. **^How much do you trust AI-based decisions in hepatology compared to human expertise?^**

- ^No trust at all - AI is unreliable in hepatology^
- ^Limited trust - AI should always be secondary to human decisions^
- ^Moderate trust - AI is useful but needs human validation^
- ^High trust - AI can make accurate recommendations comparable to human experts^

1. **^What ethical concerns do you have about AI implementation in hepatology?^** ^(Select all that apply)^

- ^AI bias and errors leading to misdiagnosis^
- ^Liability issues in AI-assisted medical decision-making^
- ^Data security and patient confidentiality risks^
- ^Lack of evidence of the benefits of Al-generated information^
- ^Ethical concerns about AI replacing human expertise in hepatology^
- ^Lack of transparency in AI decision-making models^
- ^Over-reliance on AI and reduced clinical judgment by physicians^
- ^Other (please specify)^

1. **^Would you feel comfortable relying on AI-driven recommendations in critical hepatology cases?^**

- ^Yes, AI should be used as an independent decision-making tool^
- ^Yes, but only when combined with human validation^
- ^No, AI should not be used in critical decision-making^

1. **^Do you believe AI should have a role in patient counseling and education in hepatology?^**

- ^Yes, AI can provide personalized education and support for patients^
- ^No, human interaction is essential for effective patient counseling^
- ^AI can assist but should not replace human involvement^
- ^Uncertain, AI’s role in counseling needs further research^

**^Section 7: Enhancing AI Integration in Hepatology^**

1. **^What steps should be taken to enhance trust in AI-based hepatology solutions?^** ^(Select all that apply)^

- ^Developing clear regulatory guidelines for AI in healthcare^
- ^Ensuring AI models are transparent and interpretable^
- ^Increasing hepatologists' training on AI applications^
- ^Conducting clinical trials to validate AI effectiveness in hepatology^
- ^Encouraging collaborations between AI developers and medical professionals^
- ^Implementing AI-based decision support systems with real-time feedback^
- ^Other (please specify)^

1. **^How should AI be integrated into hepatology training programs?^** ^(Select all that apply)^

- ^AI-focused courses and certifications for hepatologists^
- ^Hands-on workshops and AI simulation-based learning^
- ^Online AI learning platforms for medical professionals^
- ^Inclusion of AI education in medical school curricula^
- ^Collaboration with AI researchers to develop hepatology-specific applications^
- ^Other (please specify)^

**^Section 8: AI Use in Hepatology Research and Publishing^**

1. **^Have you used AI-powered tools in your research work?^**

- ^Yes, frequently^
- ^Yes, occasionally^
- ^No, but I am interested in using them^
- ^No, I do not see a need for AI in research^

1. **^Which AI-based applications have you used in your hepatology research? (Select all that apply)^**

- ^AI-assisted literature review and summarization^
- ^AI-powered statistical analysis and data interpretation^
- ^AI-driven image processing for histopathology and imaging studies^
- ^AI-based manuscript writing assistance^
- ^AI-driven predictive modeling for hepatology studies^
- ^Other (please specify)^

1. **^What is your primary concern regarding AI use in research and publication? (Select all that apply)^**

- ^Accuracy and reliability of AI-generated results^
- ^Ethical concerns regarding AI-assisted manuscript writing^
- ^Lack of proper validation for AI-driven research methodologies^
- ^AI bias leading to incorrect conclusions^
- ^Other (please specify)^

1. **^Do you think AI can improve the efficiency and quality of research in hepatology?^**

- ^Yes, significantly^
- ^Somewhat, but with human oversight^
- ^No, traditional methods are more reliable^
- ^Uncertain^

1. **^Would you be open to attending AI training workshops focused on its application in hepatology research?^**

- ^Yes, definitely^
- ^Maybe, if the training is relevant to my work^
- ^No, I do not see the need^

**^Conclusion^**

^Thank you for your participation. Your insights will help shape future research, policy recommendations, and training programs for AI integration in hepatology in the MENA region.^

^Contact Prof. Mohamed El-Kassas ;^ [^m_elkassas@hq.helwan.edu.eg^](mailto:m_elkassas@hq.helwan.edu.eg)^.^
