# Supplementary material for: Mapping artificial intelligence adoption in hepatology practice and research: challenges and opportunities in MENA region
Source: Front Med (Lausanne). 2025 Sep 12;12:1630831. doi: 10.3389/fmed.2025.1630831 (PMC12463891; doi:10.3389/fmed.2025.1630831)
Supplement: Supplementary file 2 [file Supplementary_file_2.docx]

**Supplementary Table 1. Thematic synthesis of hepatologists’ policy recommendations for AI adoption in hepatology training and practice in the MENA region**

| **Theme** | **Key recommendations from respondents** |
| --- | --- |
| **1. Governance and regulation** | Establish national legal and ethical frameworks; define accountability among developers, implementers, institutions, and clinicians; require disclosure of AI use in clinical research and care; develop clear approval pathways and validation standards before clinical deployment; align hospital policies with national regulations; update consent processes to reflect AI use; ensure medico legal clarity and liability allocation. |
| **2. Data governance, privacy, and security** | Enforce strict data privacy and confidentiality protections; enable secure data sharing and storage; employ anonymization; update data sharing laws; prevent misuse or unfair commercial competition; protect patient information from cyber risks; redefine protected data elements where needed. |
| **3. Technical standards and interoperability** | Standardize terminology and datasets; ensure compatibility and interoperability between AI tools and existing EHR and laboratory and imaging systems; minimize errors from manual data entry; promote common data models to support cross center use. |
| **4. Education and capacity building** | Integrate AI literacy into undergraduate and postgraduate curricula; provide regular workshops, seminars, simulation based learning, and certifications; train clinicians in practical AI skills (including basic coding where relevant); expand institutional subscriptions to educational tools; raise awareness among clinicians and trainees. |
| **5. Clinical integration and guidelines** | Incorporate AI into clinical guidelines with clear evidence thresholds; define AI’s role as decision support with clinician oversight; pilot use in high value areas such as imaging, pathology slide reading, fibrosis staging, and transplant evaluation; maintain humanization of care and preserve clinical judgment. |
| **6. Equity, access, and cost** | Improve availability and affordability of AI tools; consider subsidized or free access in public and academic settings; address the digital divide across countries and institutions; ensure suitability and cost effectiveness in low resource settings. |
| **7. Research, validation, and monitoring** | Support region specific studies and external validation on local datasets; monitor performance post deployment; reduce bias and improve generalizability; encourage journals and shared channels that facilitate AI research dissemination; expand datasets and examples for training and evaluation. |
| **8. Stakeholder engagement and implementation** | Engage physicians, patients, administrators, ministries, and regulators; ensure hospital leadership supports digital transformation; promote consensus building and collaboration between clinicians and AI developers; establish technical support and AI monitoring units; start with primary care and routine use where appropriate. |

**Abbreviations:** AI, artificial intelligence; EHR, electronic health record; MENA, Middle East and North Africa.
